# Cyberaddiction in the medical setting: A study of 45 cases

**DOI:** 10.1192/j.eurpsy.2024.828

**Published:** 2024-08-27

**Authors:** A. Ghenim, D. Brahim, I. Yaich, C. Ben Said, A. Belkahla, I. Youssef, M. Mersni, N. Mechergui, H. Ben Said, G. Bahri, M. Bani, N. Bram, N. Ladhari

**Affiliations:** ^1^Occupational pathology and fitness for work department, Faculty of medicine of Tunis, Tunis El Manar University, Charles Nicolle Hospital, TUNIS; ^2^Forensic Psychiatry department, Faculty of medicine of Tunis, Tunis El Manar University, Razi Hospital, MANOUBA; ^3^Forensic Psychiatry department, Faculty of medicine of Tunis, Tunis El Manar University, Razi Hospital, TUNIS, Tunisia

## Abstract

**Introduction:**

Internet use can become uncontrollable, leading to physical and psychological suffering and what is known as cyberaddiction.

**Objectives:**

To assess the frequency of cyberaddiction in a population of young doctors.

**Methods:**

We conducted a cross-sectional, descriptive study of a population of young doctors. We collected socio-professional and medical data using a Google Forms self-questionnaire. The Young scale was recommended for screening for cyberaddiction. A score ≥5 indicates Internet addiction. The Hospital Anxiety and Depression Scale (HAD) was adopted to reveal anxiety-depressive disorders.

**Results:**

A total of 45 physicians responded to our survey. The mean age was 29.93±4.8 years. The sex ratio (M/F) was 0.3. Participants were single in 69% of cases. Residents represented 64% of the population. Physicians were family medicine residents in 11% of cases. The mean Young’s score was 3.13±1.97/8. Cyberaddiction was noted in 24% of cases. A definite anxiety-depressive disorder was found in 6.7% and 13.3% of cases respectively. Internet addiction was significantly associated with female gender (p<0.05) and a positive HAD (A) score (p=0.03).

**Conclusions:**

According to the results of our study, cyberaddiction is common among medical staff. A preventive strategy is needed to counter the harmful effects of this addiction.

**Disclosure of Interest:**

None Declared

